# A Computed Tomography-Based Morphometric Assessment of the Foramen Lacerum in a Turkish Population Using the 3D Slicer Method

**DOI:** 10.3390/medicina61050943

**Published:** 2025-05-21

**Authors:** Merve Muslu, Ömür Karaca, Aybars Kökçe, Niyazi Acer

**Affiliations:** 1 Department of Anatomy, Faculty of Medicine, Balıkesir University, Balıkesir 10145, Türkiye; mervemuslu99@gmail.com; 2 Department of Anatomy, Faculty of Medicine, Eskisehir Osmangazi University, Eskisehir 26040, Türkiye; aybars.kokce@ogu.edu.tr; 3 Department of Anatomy, Faculty of Medicine, Sanko University, Gaziantep 27090, Türkiye; niyazi.acer@sanko.edu.tr

**Keywords:** skull base anatomy, CT imaging, foramen lacerum, vidian canal, 3D Slicer, morphometric analysis

## Abstract

*Background and Objectives*: The foramen lacerum (FL), located at the base of the skull, is generally considered the safest anatomical pathway for accessing the internal carotid artery (ICA) and the vidian canal (VC) during surgical procedures. We aimed to evaluate the morphometric characteristics of FL, VC, and related structures. *Materials and Methods:* This study utilized cranial computed tomography (CT) images obtained between 2016 and 2018 at Balıkesir University Faculty of Medicine for various clinical indications. A retrospective analysis was performed on cranial CT images from 77 patients, comprising 42 females and 35 males. The length and width of the FL, the length of the VC, and the angles formed between the VC and the pterygosphenoidal fissure and between the VC and the palatovaginal canal were measured. All measurements were performed using the three-dimensional (3D) Slicer software to ensure precision and consistency. *Results*: Males had significantly longer right and left FL lengths and left FL width than females (*p* < 0.05). No significant gender-based differences were found in VC length on either side. The angle between the VC and the pterygosphenoidal fissure was significantly larger in males (*p* < 0.05). Additionally, increased FL length and width were significantly correlated with larger angles between the VC and the pterygosphenoidal fissure in all subjects (*p* < 0.05). The anatomical variations of the FL Type 1 (normal) were identified as the most prevalent configuration across the study population. Type 2 (canal-shaped) ranked as the second most frequent variant in females, whereas Type 3 (bridged) was the second most commonly observed form in males. *Conclusions*: Preoperative identification of FL anatomical variations, which differ between individuals and sexes, may enhance the safety of skull base surgeries and minimize postoperative complications. The morphometric data presented in this study provide valuable guidance for clinicians planning interventions involving the FL and surrounding structures, and contribute valuable insights to anatomists regarding regional morphology.

## 1. Introduction

The foramen lacerum (FL) is a critical anatomical landmark in skull base surgery due to its unique location at the intersection of several key neurovascular and skeletal structures. It is a gateway to multiple anatomical regions, making it an essential anatomic structure during surgical approaches to the skull base. Surgical exposure of the FL may be required in the management of various pathological conditions involving adjacent skull base regions, such as chondrosarcomas, cavernous sinus tumors, and lesions affecting the internal carotid artery (ICA), Meckel’s cave, petrous apex, clivus, nasopharynx, eustachian tube, and jugular foramen, as described in several clinical reports [1,2,3,4].

The FL is often misinterpreted as a true foramen. The FL (normal) is a passage bordered anteriorly by the central part of the sphenoid body (corpus sphenoidale), posteriorly by the basilar part of the occipital bone, and laterally by the petrous part of the temporal bone and the carotid canal [5]. The carotid canal, which surrounds the petrous segment of the ICA, opens at the posterolateral margin of the FL and extends anteromedially along its superior aspect [6]. The ICA courses over fibrocartilaginous tissue covering the FL’s superior surface, and it contacts the body of the sphenoid bone. The segment of the ICA located immediately superior to the FL is known as the lacerum segment [6,7]. After passing through the FL region, the ICA bends ventrally and inferiorly to enter the cavernous sinus. The trigeminal ganglion is situated on the posterior-lateral side of the FL. Additionally, the vidian nerve formed by the greater and deep petrosal nerves, enters the vidian canal (VC) anterior to the FL. The VC runs anteriorly from the anterior border of the FL, passing through the sphenoid sinus floor and ultimately terminating in the pterygopalatine fossa. The FL is situated in the posterolateral direction of the VC, marking a critical junction in the anatomy of the skull base [4,8,9]. For example, one study has stated that in endoscopic endonasal approaches for safely accessing the FL, the vidian nerve should be traced from the pterygopalatine fossa to the FL [4]. These anatomical relationships are significant as they highlight the close spatial proximity among the FL, VC, ICA, and neighboring skull base structures [10].

Although the FL does not transmit major anatomical structures, it allows the passage of small meningeal branches from the ascending pharyngeal artery and emissary veins. These emissary veins traverse the fibrocartilaginous tissue of the FL, establishing a direct connection between the cavernous sinus and the pterygoid venous plexus [11,12,13]. Clinically, this venous pathway could serve as a route for migrating pathological agents from extracranial regions to intracranial structures, highlighting the clinical importance of the FL in these venous connections.

Historically, anatomist Wenzel Gruber identified and described the FL in 1869 [14]. Its anatomical significance has been further explored through various dissections, dry skull studies, and radiological imaging techniques [4,15,16]. Despite extensive research, the FL remains a site of significant anatomical variation, including differences in its size, shape, location, and the neurovascular structures that pass nearby [6,15,17]. These variations make the FL challenging surgical procedures, particularly in endonasal endoscopic approaches where precision and safety are paramount [1,4,18]. As such, detailed preoperative planning is crucial to minimize the risk of inadvertent injury to the surrounding structures, particularly the ICA, trigeminal ganglion, and the vidian nerve.

Despite the valuable insights gained from anatomical dissections and radiological imaging techniques, such as computed tomography (CT) scans and magnetic resonance imaging (MRI), there remains a need for standardized and practical measurement methods to accurately assess the anatomy of the FL in individual patients [1,2,4,5]. CT imaging, in particular, is invaluable for delineating cranial bone structures. Three-dimensional (3D) imaging technologies, such as the 3D Slicer software https://www.slicer.org (accessed on 1 August 2023), have been widely adopted in neurosurgery for their ability to provide detailed 3D visualizations of the skull base [19,20,21]. This software is handy for segmenting and visualizing complex anatomical regions, such as the FL. It offers surgeons a clear understanding of the anatomical relationships that may influence surgical approaches.

This study aims to conduct a morphometric analysis of the FL using CT imaging and 3D visualization techniques. By assessing the FL’s anatomical variations and its relationship with the VC, the research seeks to provide valuable insights into safe surgical approaches while contributing to the broader anatomical literature. A comprehensive understanding of the FL’s morphology and its variations will enhance preoperative planning and improve patient outcomes in skull base surgeries.

## 2. Materials and Methods

### 2.1. Study Population

This retrospective cross-sectional study analyzed 3D cranial CT images from 77 individuals, consisting of 42 females and 35 males, aged between 18 and 80 years, with a mean age of 47.16 ± 15.94 years. The cranial CT scans were obtained from the Picture Archiving and Communication System (PACS) at Balıkesir University Hospital, covering scans taken between 2016 and 2018. These scans were acquired initially for routine diagnostic evaluations for various clinical reasons, such as ear pain, eye discomfort, and headaches. This study included CT images from participants with no evidence of skull base pathologies, sphenoid bone fractures, or sphenoid sinus abnormalities. All CT images were meticulously reviewed and analyzed by a neuroradiologist with over 15 years of experience in neuroradiology. CT scans with poor image quality were excluded from this study to ensure the accuracy and reliability of the measurements.

This study received approval from the Clinical Research Ethics Committee of Balıkesir University (approval date: 11 October 2023; approval number: 2023/134). All procedures in this study were conducted according to the ethical standards in the Declaration of Helsinki.

### 2.2. Cranial CT Protocol

Cranial CT images were acquired using a 64-slice CT scanner (Aquillon 64, Toshiba, Otawara, Japan), which provides high-resolution imaging suitable for detailed analysis of cranial structures. The images were obtained with a slice thickness of 1 mm to ensure fine anatomical detail and allow for precise morphometric measurements. Imaging parameters were set to 120 kV (kilovolts) and 200 mAs (milliampere-seconds), optimal settings for maximizing image clarity. The pixel spacing for the CT scans was set to 0.3 × 0.3 mm. The axial CT images were then reconstructed into 3D models using the 3D Slicer software, allowing for comprehensive visualization and analysis of the skull base anatomy.

### 2.3. Morphometric Analysis with 3D Slicer

3D Slicer is a free, open-source medical image computing and visualization software application https://www.slicer.org (accessed on 1 August 2023) [19]. The 3D Slicer method used in this study has been extensively validated in previous research, confirming its accuracy, reproducibility, and effectiveness for investigations of the skull base [21,22]. Our study processed and analyzed cranial CT images using 3D Slicer version 5.3.1. Intra-rater reliability was assessed by having the same evaluator perform the measurements twice at different time points. The average of the two measurements was used for analysis.

**Figure 1:**
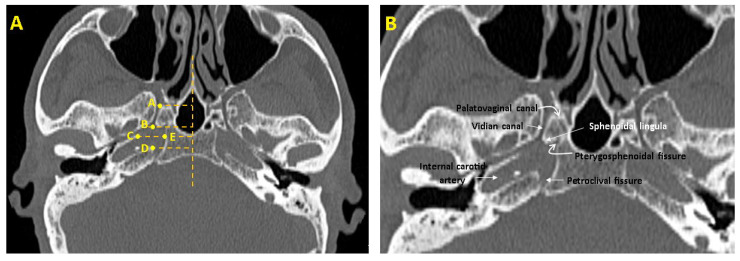
Anatomical landmarks used for morphometric measurements in the axial plane. (**A**) A. Anterior end of the vidian canal opening into the pterygopalatine fossa; B. Posterior end of the vidian canal opening into the foramen lacerum; C. Line drawn from the apex of the petrous part of the temporal bone to the midsagittal plane; D. Line drawn from the apex of the petrous part of the temporal bone and the petroclival fissure to the midsagittal plane; E. The point where the line extending from point C to the midsagittal plane intersects the corpus sphenoidale. (**B**) Anatomical structures used for measurements on CT images.

Morphometric measurements were conducted using predefined anatomical reference points to ensure consistency across all subjects. These landmarks were selected based on their high reproducibility, clear visibility in axial CT images, and relevance in previously published studies on skull base anatomy (Table 1) [1,4,6,15,23,24]. For instance, the base of the sphenoidal lingula, the pterygosphenoidal fissure, and the petroclival fissure were identified by established anatomical descriptions (Figure 1) [1,4,23,24]. All measurements were performed in a standardized axial plane for each individual. For each participant, the axial section in which the vidian canal was most clearly visible was identified, and all measurements were performed on this selected plane (Figure 1). By consistently using the most optimal section for each individual, measurement accuracy and reproducibility were ensured throughout the study.

**Figure 2:**
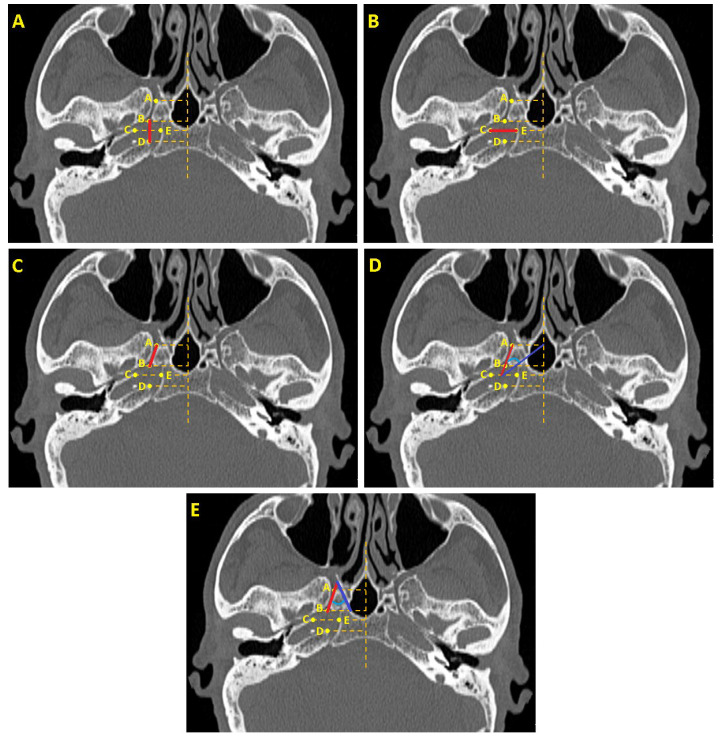
(**A**) Length of the foramen lacerum (indicated by the red line). (**B**) Width of the foramen lacerum (indicated by the red line). (**C**) Length of the vidian canal (indicated by the red line). (**D**) Angle between the vidian canal (indicated by red line) and the pterygosphenoidal fissure (indicated by blue line). (**E**) Angle between the vidian canal (indicated by red line) and the palatovaginal canal (indicated by blue line).

We assessed the following structures: the length and width of the FL, the length of the VC, the angles between the VC and pterygosphenoidal fissure, and the angles between the VC and the palatovaginal canal (Figure 2) (Table 1).

**Figure 3:**
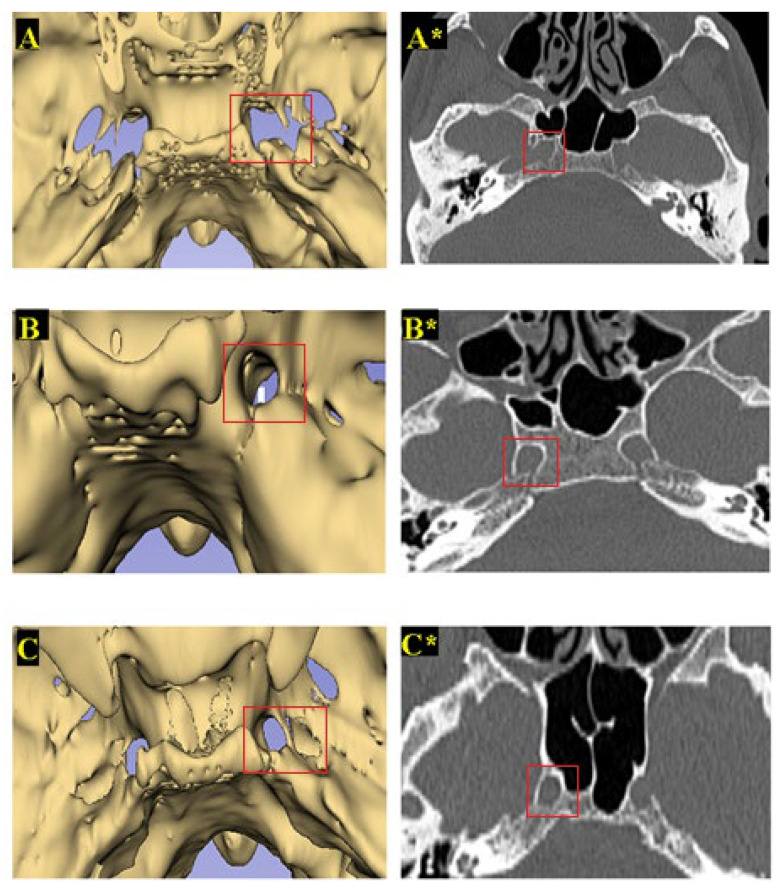
Type 1. Normal FL (**A**,A*); Type 2. Canal-shaped FL (**B**,B*); Type 3. Bridged FL (**C**,C*).

Based on the criteria and definitions established in previous studies [5,6,17], the FL was classified into three types according to morphological characteristics observed in anatomical assessments. Type 1 (normal) represents a normal opening with standard configuration. Type 2 is a canal-shaped FL, characterized by the formation of a distinct bony channel. Type 3 refers to a bridged FL, which results from incomplete ossification of the FL and the development of a bony bridge (Figure 3).

### 2.4. Statistical Analysis

Statistical analyses were performed using the Statistical Package for the Social Sciences (SPSS) version 16.0 for Windows (SPSS Inc., Chicago, IL, USA). Sample size estimation was conducted using power analysis. Assuming a large effect size (Cohen’s d = 0.8), a statistical power of 95% (1 − β = 0.95), and a significance level of 5% (α = 0.05) for a one-tailed independent samples *t*-test, the required minimum sample size was calculated to be 70 participants. In this study, measurements were obtained from 77 participants, exceeding the minimum requirement and thereby ensuring adequate statistical power.

The normality of the data was assessed using the Shapiro–Wilk test to determine the appropriateness of parametric methods. Group comparisons, such as between male and female participants or across different FL types, were performed using independent samples *t*-tests to evaluate statistically significant differences in measured parameters. Pearson’s correlation coefficient was calculated to explore relationships between normally distributed variables. This method allowed for the assessment of linear relationships between two continuous variables, providing insight into the strength and direction of their association. A *p*-value of less than 0.05 was considered statistically significant for all tests. All statistical analyses were conducted with a confidence level of 95%.

## 3. Results

### 3.1. FL and VC Measurements

Thisstudy included 42 females and 35 males, with ages ranging from 18 to 81 years. The mean age was 46.79 ± 14.60 years for females and 47.60 ± 17.61 years for males, with no statistically significant difference between the two groups (*p* = 0.825). Morphometric parameters were analyzed by gender to identify specific differences, as shown in Table 2. According to the independent samples *t*-test results, males had significantly longer right and left FL lengths than females (*p* < 0.05). In addition, the left FL width was significantly greater in males than in females (*p* = 0.030), whereas no significant difference was observed in the right side. No significant gender-based differences were found in VC length on either side. However, the bilateral angles between the VC and the pterygosphenoidal fissure were significantly larger in males than in females (*p* = 0.001). There were no significant differences between men and women in the angle of the VC-palatovaginal canal.

When comparing the right and left sides in females, the width of the right FL was significantly greater than that of the left (*p* = 0.025) (Table 3). Similarly, the VC-pterygosphenoidal angle on the right side was significantly larger than on the left (*p* = 0.024). In females, no other parameters exhibited significant differences between the two sides. (*p* > 0.05). In male subjects, a significant asymmetry was found only in VC length, which was significantly greater on the left side than on the right (*p* = 0.022) (Table 4).

### 3.2. Classification of FL Types

Analysis of FL types revealed that the right FL was classified as Type 1 in 70% of participants, Type 2 in 15.6%, and Type 3 in 14.3%, whereas the left FL was classified as Type 1 in 72.7%, Type 2 in 11.7%, and Type 3 in 15.6% (Table 5).Among the identified FL types, Type 1 (normal) was the most prevalent in both genders, followed by Type 2 (canal-shaped) in females and Type 3 (bridged) in males (Table 5).

### 3.3. Correlation Analysis

**Figure 4:**
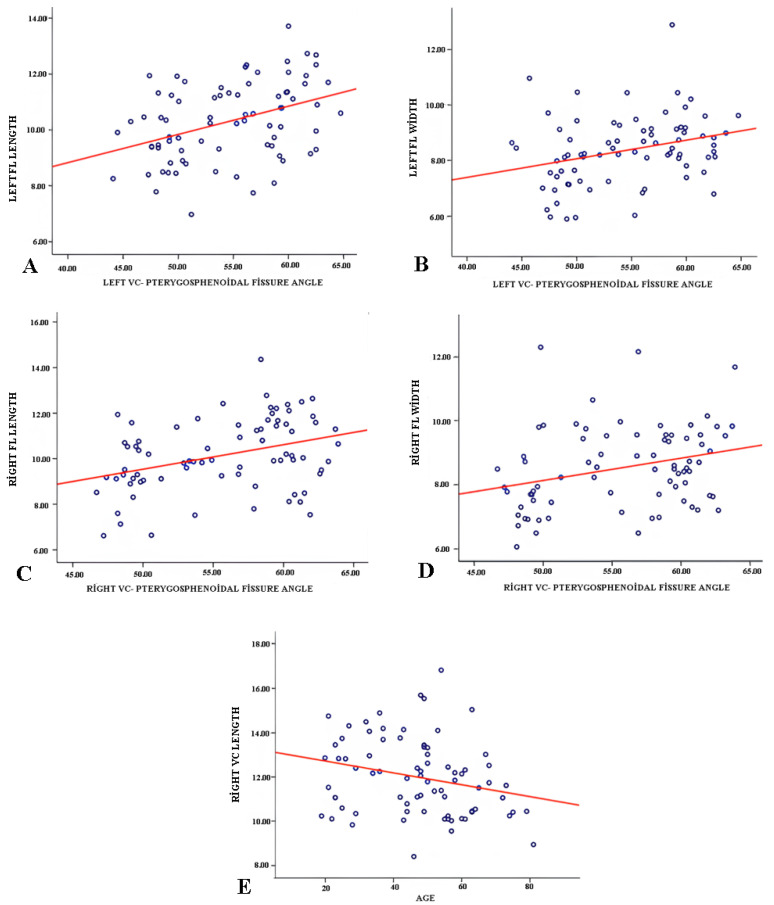
Dot plot of the correlation analysis (**A**) left foramen lacerum (FL) length and left VC-pterygosphenoidal fissure angle (*r* = 0.393, *p* < 0.05); (**B**) left FL width and left VC-pterygosphenoidal fissure angle (*r* = 0.294, *p* < 0.05); (**C**) right FL length and right VC-pterygosphenoidal fissure angle (*r* = 0.357, *p* < 0.05); (**D**) right FL width and right VC-pterygosphenoidal fissure angle (*r*= 0.285, *p* < 0.05); (**E**) right VC length and age (*r* = −0.248, *p* < 0.05).

Correlation analysis revealed a statistically significant negative relationship between age and the length of the right VC (r = –0.248, *p* = 0.029), indicating that the length of the right VC tends to decrease with increasing age (Figure 4E). No significant correlation was found between age and other morphometric measurements. Additionally, increased FL length and width were significantly correlated with larger angles between the VC and the pterygosphenoidal fissure (*p* < 0.05) (Figure 4). This suggests that the angle between the VC and the pterygosphenoidal fissure increases as the FL becomes longer and broader.

## 4. Discussion

The FL at the base of the skull is of critical importance for neurosurgical approaches, and understanding its variability is essential for endoscopic endonasal skull base surgery. In our study, males exhibited significantly greater FL lengths and wider left FL than females. Moreover, increased FL length and width were significantly associated with larger angles between the VC and the pterygosphenoidal fissure. Type 1 FL (normal) was the most common variant, observed on 70% of the right and 73% of the left sides.

Previous studies on dry skulls have provided essential baseline data on the dimensions of the FL, reporting mean lengths ranging from 3.0 mm to 16.0 mm and mean widths between 2.0 mm and 7.0 mm [6,15,25]. In a study by Naeem and Farid (2019), the analysis of 100 dry skulls from an Egyptian population revealed that the mean length of the right FL was 10.89 mm, while the left FL measured 10.88 mm [6]. Chaudhary et al. (2023) reported variations in FL length within an Indian population. In their study, males exhibited a longer right FL (6.24 mm), while females showed a longer left FL (5.40 mm). They also found that 70.7% of males and 52.4% of females had symmetric FLs on both sides [15]. Our 3D brain CT imaging analysis in the Turkish population demonstrated no asymmetry in FL length between the right and left sides in both groups. However, when considering FL width, the right FL was wider than the left in females. Few studies have investigated the morphology of the FL using cranial CT imaging [4,26]. Storey et al. evaluated the FL at the external cranial base using angiographic images and reported a mean FL length of 6.44 mm and a width of 4.56 mm. Notably, the study population consisted exclusively of African-American females with stenotic FL. The authors noted that when these stenotic cases were excluded, the mean FL length and width increased to 8.14 mm and 5.13 mm, respectively [26]. Ossification of fibrocartilaginous tissue can lead to the narrowing of the FL [15]. When this occurs, combined with natural variations in the size of the foramen, it may result in compression of the neurovascular bundle, which includes the deep petrosal nerve, greater petrosal nerve, and vidian nerve. In particular, the smaller dimensions of the FL observed in females may increase the risk of neurovascular compression. This highlights the necessity for individualized and site-specific surgical planning.

Variations in the dimensions of the FL may also be attributed to its irregular shape and morphological differences. With recent advancements in skull base surgery and middle cranial fossa approaches, understanding the morphometric variations of the FL across different geographical regions, sexes, and populations has gained increasing significance. In 2020, Singh and Kumar examined the morphological types of FL in Indian people using dry skulls and classified them into three distinct types. Type 1 was considered the normal form, Type 2 was described as a circular opening due to complete obliteration of the elongated portion of the FL, and Type 3 was characterized by incomplete ossification of the FL, resulting in the formation of a bony bridge [17]. According to their findings, complete obliteration of the FL (Type II-canal-shaped) was observed in 19.2% of cases, while incomplete ossification (Type III-bridged) was identified in 26.9% [17]. In a study conducted by Demir in Turkish people, the position of the FL relative to the medial pterygoid process (MPP) was classified into three types. Type I is defined as the FL being located posteromedial to the MPP and anterior to the internal opening of the carotid canal. Type II is a thin canal completely closed by the canalis caroticus and positioned posteromedial to the MPP. Type III is characterized by the FL extending to both sides of the MPP, covering a larger area, and expanding toward the foramen ovale [5]. According to Demir’s findings, Type I FL was the most frequently observed configuration on the right side, occurring in 55.3% of cases. Conversely, on the left side, the most common type was Type II (canal-shaped), identified in 44.6% of cases [5]. Nayak (2019) suggested that smaller left FLs often result from bony extensions of the sphenoid body; however, no formal classification system was provided [16]. In our study, some FLs were laterally bound by bone tissue, forming canal-like or bridged configurations. Based on these variations and the literature, which is described above, we categorized FL morphology into three types: Type 1 (normal), Type 2 (canal-shaped), and Type 3 (bridged). Type 1 was the most prevalent in 70% of right FLs and 73% of left FLs. Notably, Type 2 FL was more common in females, whereas Type 3 FL was more frequently observed in males. Our findings are consistent with those of Demir et al., who reported FL types in the Turkish population. To the best of our knowledge, our study is the first to present a comprehensive classification of FL morphology and to visualize these variations using 3D imaging. Interestingly, although canal-type and bridged-type FLs exhibited indistinguishable appearances on conventional CT scans, 3D reconstruction with 3D Slicer delineated their structural differences. This finding underscores the superiority of 3D imaging in identifying subtle anatomical variations that may be misinterpreted or overlooked in standard two-dimensional imaging. Therefore, 3D evaluation should be considered a valuable tool in preoperative planning, especially in complex skull base anatomy cases.

A thorough understanding of FL’s morphology and spatial relationship with adjacent anatomical structures is essential for identifying safe and effective surgical pathways during skull base procedures. The FL is medial and superior to the VC; anatomically, the VC opens anteriorly into the pterygopalatine fossa and posteriorly at the FL. One key step in accessing the FL involves tracing the posterior pathway of the vidian nerve as it exits the pterygopalatine fossa [4]. Drilling the inferior 180° of the vidian canal has become a well-established technique to help identify the Lacerum segment of the ICA [10]. This step provides a critical orientation point for surgical dissection and facilitates a controlled neurovascular approach to the FL, thereby minimizing the risk of inadvertent injury to surrounding neurovascular structures. Additionally, Mato et al. (2015) emphasized that the length of the VC is a crucial factor influencing the extent of bone drilling required during surgical procedures [27]. Their study reported mean VC lengths of 14.4 mm on the right side and 14.7 mm on the left. Similarly, other studies have reported VC lengths ranging from 10 to 19 mm [9,23,28]. A study on the Turkish population using CT imaging reported mean VC lengths of 13.09 mm on the right side and 13.01 mm on the left, with no statistically significant gender-based differences [9]. In contrast, our study demonstrated slightly lower mean values, with VC lengths measuring 11.9 mm on the right and 12.1 mm on the left. Similarly, no significant gender-related differences were observed in our sample. Although both studies were conducted within the same population, the observed variation may be attributed to methodological differences, sample size discrepancies, or underlying population heterogeneity. Notably, our findings revealed that the left VC was significantly longer in males than the right, indicating asymmetry in VC morphology among males. Therefore, this study contributes to the existing literature by emphasizing gender-specific and side-dependent anatomical variations within the Turkish population.

Recent studies have highlighted the pterygosphenoidal triangle’s importance as a novel anatomical landmark in endoscopic endonasal skull base surgery. This triangle is delineated by the pterygosphenoidal fissure medially and the vidian nerve laterally, with its apex formed by the pterygoid tubercle (sphenoidal lingula) leading to the FL [1]. The pterygosphenoidal fissure is one of the three fissures that constitute the FL. Because it is located most anteriorly, it is ideally suited as a surgical landmark when performing anterior and medial approaches, in the endoscopic endonasal approach. Wang et al. (2019) provided a detailed investigation of the endonasal surgical anatomy, highlighting that the medial-to-lateral trajectory of the endonasal approach offers direct access to the FL, enabling safe and practical exposure of both the FL and the lacerum segment of the ICA [4]. Drilling begins at the floor of the sphenoid sinus and the pterygoid process. Once the floor of the sphenoid sinus and the vaginal process are removed, the medial surface of the pterygosphenoidal fissure becomes visible. Tracing the course of the pterygosphenoidal fissure simplifies access to the FL. The fibrous tissue within the pterygosphenoidal fissure is a reliable anatomical landmark during surgery [1,4]. Therefore, understanding the trajectory and angle of the fissure is crucial for surgical success. The pterygosphenoidal fissure consistently converges with the posterior end of the vidian canal at an angle of approximately 45°, with its posterolateral end pointing directly toward the FL [4]. As the angle between the vidian canal and the pterygosphenoidal fissure corresponds to the anterior portion of the FL, precisely identifying this angle is essential during preoperative planning. Wang et al. (2019) measured this angle in 32 individuals, finding mean values of 45.46º on the right and 45.39º on the left, with no significant difference between sides [4]. In comparison, our study observed larger mean angles of 55.70º on the right and 54.48º on the left, with males exhibiting wider angles. Furthermore, our findings revealed that FL length and width increases correlated with larger VC-pterygosphenoidal fissure angles. Accordingly, the degree of the angle may be influenced by the length and width of the FL across different populations and genders.

Along with the anatomical structures mentioned earlier, the palatovaginal canal is a reliable and consistent landmark for identifying the VC during endoscopic endonasal and transpterygoid surgical approaches [23]. Also referred to as the palatosphenoidal or pharyngeal canal, the palatovaginal canal is a short bony tunnel formed by the articulation of the vaginal process of the sphenoid bone and the sphenoidal process of the palatine bone [1]. Anatomically, the palatovaginal canal and its accompanying artery are situated at the base of the pterygosphenoidal triangle. It extends anteriorly along the body of the pterygoid process and lies medial to the VC [8]. The proximity of the VC and the palatovaginal canal poses a challenge in distinguishing these structures during endoscopic procedures. Despite its clinical significance, few studies have investigated this relationship.

In our study, we measured the mean angle between the VC and the palatovaginal canal, finding it to be 50.49° on the right side and 50.68° on the left side. We observed that males exhibited wider angles than females. In contrast, Kurt et al. (2020) reported larger angles in a Turkish population, with mean values of 66.12° on the left side and 65.87° on the right side, and they found no significant gender differences [9]. Pinheiro-Neto et al. (2012) documented slightly smaller mean angles of approximately 48° in a CT-based study involving 15 individuals [29]. In contrast, another study reported mean angles of the VC-palatovaginal canal as large as approximately 60.99° in an Indian population [23]. These findings highlight the importance of considering population and gender-specific anatomical variations when evaluating the relationship between the VC and the palatovaginal canal.

Although this study obtained essential results, it had some limitations. One limitation is the relatively small sample size drawn from a single center within the Turkish population. This limitation may reduce the generalizability of the findings to broader populations. A larger sample size would allow for more precise morphometric evaluations and robust statistical analyses. Additionally, although the current study focused on morphometric parameters, it did not evaluate the functional effects of FL and VC variations in surgical procedures or their potential relationships with clinical outcomes. Therefore, future studies should consider including larger cohorts from multiple centers, age-related changes, and diverse ethnic backgrounds to enhance the external validity and applicability of the results. Additionally, investigating the functional implications of FL variations, particularly their impact on surgical approaches and outcomes, would provide valuable insights for clinical practice.

## 5. Conclusions

Our study highlights significant anatomical variations in the FL and its surrounding structures within the Turkish population. We observed notable gender-specific differences in the FL’s length, width, and morphological types, emphasizing the importance of accounting for individual anatomical variations, particularly in surgical planning. Moreover, our findings contribute to the existing anatomical literature by enhancing the understanding of FL morphology, which may have important implications for medical education and future research in neuroanatomy and related disciplines.

## Figures and Tables

**Table 1:** Anatomical landmarks for morphometric measurements in the axial plane.

Parameters	Definitions of Landmarks (Figure 1)
FL length(mm)	The distance between the posterior end of the VC opening into the FL and the petrous apex of the temporal bone (B–D).
FL width (mm)	The distance between the temporal bone’s petrous apex and the sphenoid bone’s corpus (C–E).
VC length (mm)	The distance between the anterior end of the VC opening into the pterygopalatine fossa and the posterior end of the VC opening into the FL (A,B).
VC—pterygosphenoidal fissure angle (°)	The angle formed between the VC and the oblique line drawn from the pterygosphenoidal fissure.
VC—palatovaginal canal angle (°)	The angle formed between the VC and the oblique line drawn from the palatovaginal canal.

FL: foramen lacerum VC: vidian canal.

**Table 2:** Gender-specific differences of morphometric parameters as determined via independent samples *t*-test.

Parameters	Gender	Mean ± SD	SE	*p*-Value
Right FL length (mm)	Female	9.76 ± 1.52	0.23	0.017 *
	Male	10.62 ± 1.53	0.25	
Left FL length (mm)	Female	9.97 ± 1.36	0.21	0.022 *
	Male	10.69 ± 1.34	0.22	
Right FL width (mm)	Female	8.44 ± 1.23	0.19	0.516
	Male	8.63 ± 1.34	0.22	
Left FL width (mm)	Female	8.09 ± 1.12	0.17	0.030 *
	Male	8.70 ± 1.30	0.22	
Right VC length(mm)	Female	12.11 ± 1.70	0.26	0.508
	Male	11.84 ± 1.75	0.29	
Left VC length (mm)	Female	12.36 ± 1.40	0.21	0.949
	Male	11.84 ± 1.90	0.32	
Right VC-pterygosphenoidal fissure angle (°)	Female	53.80 ± 5.32	0.82	0.001 *
	Male	57.97 ± 4.16	0.70	
Left VC-pterygosphenoidal fissure angle (°)	Female	52.55 ± 5.62	0.86	0.001 *
	Male	57.01 ± 4.15	0.70	
Right VC-palatovaginal canal angle (°)	Female	50.04 ± 2.27	0.35	0.154
	Male	51.03 ± 3.71	0.62	
Left VC-palatovaginal canal angle (°)	Female	50.25 ± 2.27	0.35	0.139
	Male	51.20 ± 3.25	0.54	

* represents a significant difference (*p* < 0.05). FL: foramen lacerum, VC: vidian canal, SD: standard deviation, SE: standard error.

**Table 3:** Comparison of right and left-sided FL and VC measurements in females.

Parameters	Right (Mean ± SD)	Left (Mean ± SD)	Mean Difference	t	df	*p*-Value
FL length (mm)	9.76 ± 1.53	9.97 ± 1.37	−0.21	−1.27	41	0.212
FL width (mm)	8.44 ± 1.24	8.09 ± 1.13	0.35	2.34	41	0.025 *
VC length (mm)	12.11 ± 1.71	12.36 ± 1.41	−0.25	−1.19	41	0.242
VC–pterygosphenoidal angle (°)	53.81 ± 5.33	52.56 ± 5.63	1.25	2.34	41	0.024 *
VC–palatovaginal canal angle (°)	50.04 ± 2.27	50.26 ± 2.27	−0.22	−0.63	41	0.530

* represents a significant difference (*p* < 0.05). FL: foramen lacerum, VC: vidian canal, SD: standard deviation.

**Table 4:** Comparison of right and left-sided FL and VC measurements in males.

Parameter	Right (Mean ± SD)	Left (Mean ± SD)	Mean Difference	t	df	*p*-Value
FL length (mm)	10.62 ± 1.53	10.70 ± 1.35	−0.08	−0.54	34	0.591
FL width (mm)	8.63 ± 1.34	8.71 ± 1.30	−0.07	−0.37	34	0.712
VC length (mm)	11.85 ± 1.75	12.36 ± 1.91	−0.54	−2.39	34	0.022 *
VC-pterygosphenoidal angle (°)	57.97 ± 4.17	57.02 ± 4.15	0.96	1.48	34	0.148
VC–palatovaginal canal angle (°)	51.03 ± 3.72	51.20 ± 3.25	−0.17	−0.25	34	0.807

* represents a significant difference (*p* < 0.05). FL: foramen lacerum, VC: vidian canal, SD: standard deviation.

**Table 5:** Classification of FL types.

FL Types	Total (%/n)	Female (%/n)	Male (%/n)
Right FL			
Type 1 (Normal)	%70.1 (54)	%64.3 (27)	%77.1 (27)
Type 2 (Canal-shaped)	%15.6(12)	%26.2 (11)	%2.9 (1)
Type 3 (Bridged)	%14.3 (11)	%9.5 (4)	%20.0 (7)
Left FL			
Type 1 (Normal)	%72.7 (56)	%73.8 (31)	%71.4 (25)
Type 2 (Canal-shaped)	%11.7 (9)	%16.7 (7)	%5.7 (2)
Type 3 (Bridged)	%15.6 (12)	%9.5 (4)	%22.9 (8)

FL: foramen lacerum.

## Data Availability

We confirm that the main data supporting this study’s findings are available within the article, and any other additional data are available on request.

## Abbreviations

The following abbreviations are used in this manuscript:

AP	Antero-posterior
CT	Computed tomography
FL	Foramen lacerum
ICA	Internal carotid artery
MRI	Magnetic resonance imaging
PACS	Picture archiving and communication system
SPSS	Statistical Package for the Social Sciences
VC	Vidian canal
3D	Three-dimensional
